# DEM-TACE as the initial treatment could improve the clinical efficacy of the hepatocellular carcinoma with portal vein tumor thrombus: a retrospective controlled study

**DOI:** 10.1186/s12885-022-10361-5

**Published:** 2022-11-30

**Authors:** Junwei Chen, Lisha Lai, Junyang Luo, Haofan Wang, Mingan Li, Mingsheng Huang

**Affiliations:** 1 Department of Interventional Radiology, the Third Affiliated Hospital of Sun Yat-Sen University, Tianhe Road 600#, Tianhe District, Guangzhou, 510630 Guangdong China; 2 Department of Radiology, Guangzhou First People’s Hospital, School of Medicine, South China University of Technology, Guangzhou, Guangdong 510030 China

**Keywords:** Transarterial chemoembolization, Drug-eluting microsphere, Hepatocellular carcinoma, Portal vein tumor thrombosis

## Abstract

**Background:**

Conventional-transarterial chemoembolization (C-TACE) was proven to improve overall survival (OS) in hepatocellular carcinoma (HCC) patients with portal vein tumor thrombus (PVTT), drug-eluting microsphere-TACE (DEM-TACE) was supposed to provide more benefit than C-TACE in this respect.

**Purpose:**

To compare the safety and efficacy between DEM-TACE and C-TACE as the initial treatment in HCC patients with PVTT and to identify prognostic factors of OS.

**Methods:**

The medical records of advanced HCC patients with PVTT who underwent DEM-TACE or C-TACE as the initial thearpy from September 2015 with mean follow-up time 14.9 ± 1.2 (95% CI 12.6–17.2) months were retrospectively evaluated. A total of 97 patients were included, 49 patients in the DEM-TACE group and 48 in the C-TACE group. Adverse events (AEs) related to TACE were compared. Tumor and PVTT radiologic response, time to tumor progression (TTP) and OS were calculated and compared in both groups.

**Results:**

Patients in DEM-TACE group had a better radiologic response (Tumr response: 89.8% vs. 75.0%; PVTT response: 85.7% vs. 70.8%; overall response: 79.6% vs. 58.3%, *P* = 0.024) and longer TTP (7.0 months vs. 4.0 months, *P* = 0.040) than patients in C-TACE group. A lower incidence of abdominal pain was found in the DEM-TACE group than in C-TACE group (21 vs. 31, *P* = 0.032), but there were no significant differences between DEM-TACE and C-TACE patients in any other AEs reported. When compared to C-TACE, DEM-TACE also showed significant OS benefits (12.0 months vs. 9.0 months, *P* = 0.027). DEM-TACE treatment, the absence of arterioportal shunt (APS), lower AFP value and better PVTT radiologic response were the independent prognostic factors for OS in univariate/multivariate analyses, which provided us with a guide for better patient selection.

**Conclusions:**

Based on our retrospective study, DEM-TACE can be performed safely and might be superior to C-TACE as the initial treatment for HCC patients with PVTT.

**Trial registration:**

Retrospectively registered.

## Introduction

Hepatocellular carcinoma (HCC) is one of the most common cancers with poor survival outcomes worldwide [1, 2]. Portal vein tumor thrombus (PVTT) occurs in up to 44% of patients with HCC at the time of death and approximately 10%-40% of patients at the time of diagnosis [3, 4]. The presence of PVTT has a strong association with prognosis with a short median survival time (2–4 months) [4]. Furthermore, PVTT also limits treatment options, including radical treatments, such as liver transplantation and curative resection, and the optimal treatment for advanced HCC patients with PVTT remains largely controversial [1].

The Barcelona Clinic Liver Cancer (BCLC) group recommended system therapy (include tyrosine kinase inhibitor-TKI and immune checkpoint inhibitors-ICIs) as a standard therapy for patients with advanced HCC (BCLC stage C), including patients with PVTT [1, 5, 6]. However, survival benefits from systemic therapy among patients with advanced-stage HCC patients with PVTT are poor[7, 8]. Several studies have demonstrated that conventional transarterial chemoembolization (C-TACE) is a palliative treatment for advanced HCC patients and that it could improve survival compared to sorafenib therapy [9, 10]. However, repeating C-TACE is limited due to decreased liver function and the resulting diminished efficacy.

The advent of drug-eluting microsphere-TACE (DEM-TACE) represents an advanced technology, as these delivery systems slowly release chemotherapeutic drugs into HCC tissues, consequently improving safety and efficacy compared with C-TACE [11–13]. Some studies indicated that DEM-TACE could decrease the number of TACE cycles and improve the early tumor response rate compared with C-TACE [14, 15]. The survival benefit of DEM-TACE has also been reported in previous study [11]. However, the significance of DEM-TACE in HCC patients with PVTT has not been reported. Therefore, this retrospective study was conducted to evaluate the safety and efficacy of DEM-TACE in advanced HCC with first-, second- or lower-order portal vein tumor thrombus compared with C-TACE.

## Materials and methods

### Study design and population

This retrospective study was approved by the ethics committee of the third affiliated hospital of Sun Yat-Sen University, and it conformed to the standards of the Declaration of Helsinki ([2021]02–288-01). Due to the retrospective nature of the study, the Institutional Review Board of the third affiliated hospital of Sun Yat-Sen University waived the need for written informed consent. We reviewed the electronic medical records of 372 advanced HCC patients with PVTT who accepted DEM-TACE or C-TACE as the initial therapy from September 2015 to August 2017 at the third Affiliated Hospital of Sun Yat-sen University, Guangzhou, China. The choice of TACE has been made on a case-to-case basis by the multi-disciplinary treatment board (consisting of interventional radiologists, medical oncologists and liver surgeons), and after in-depth discussion with the patient himself/herself. Before the initial TACE, the interventional radiologists would ask patients to choose from either DEM-TACE or C-TACE after detailed introduction of each technique and informed consent forms for DEM-TACE or C-TACE would be required to be signed. The diagnosis of HCC was based on the criteria of the European Association for the Study of the Liver (EASL) [1]. The presence of PVTT was confirmed that the detection of the enhancement of an intraluminal mass expanding portal vein (first-, second- or lower-order portal vein) on the arterial phase and a low-attenuation, intraluminal mass on the portal phase on three-phase dynamic CT/MR images.

The eligibility criteria were as follows: (a) imaging or pathological diagnosis of unresectable hepatocellular carcinoma; (b) Child–Pugh class A or B, and Eastern Cooperative Oncology Group (ECOG) performance status of 0–2; (c) presence of PVTT within 7 days before TACE; (e) no previous treatment. The exclusion criteria were as follows: (a) PVTT invade the main portal vein; (b) acceptance of surgery, liver transplantation or local–regional therapies (radiofrequency ablation, radioactive seed implantation, etc.); (c) acceptance of intra-arterial chemoinfusion; (d) other serious medical comorbidities; and (e) contraindications to lobaplatin, doxorubicin, lipiodol or TACE procedures.

### C-TACE and DEM-TACE procedures

TACE was performed using a 5-F RH catheter (Cook, Bloomington, USA) or a Cobra catheter (Cook, Bloomington, USA) and a 2.4F microcatheter (Renegade, Boston Scientific, USA; Master PARKWAY HF, Asahi, Japan; Merit Maestro Microcatheter, Merit Medical, USA) superselectively towards the tumor-feeding arteries, depending on the tumor distribution and hepatic functional reserve. Lobaplatin at a concentration of 0.5 mg/mL was infused into the tumor feeding arteries superselectively at a rate of 5 mL/min, and the total amount of lobaplatin (20 to 50 mg) depended on the patient’s body weight in the C-TACE group and DEM-TACE group. In patients with arterioportal shunt (APS), embolization using 300–700 µm Embosphere microspheres (Merit Medical, USA), which were diluted two times with contrast medium, was performed superselectively to occlude the shunt before chemoinfusion in both the C-TACE group and DEM-TACE group.

For the DEM-TACE group, 30–60 µm or 50–100 µm HepaSphere microspheres (Merit Medical, USA) loaded with 30–50 mg doxorubicin hydrochloride was injected into the tumor-feeding artery superselectively. For the C-TACE group, an emulsion of 2–20 mL lipiodol (Lipiodol Ultrafluide, Guerbet, Aulnay-Sous-Bois, France) with 20–60 mg doxorubicin hydrochloride (Pfizer, New York, USA) was also injected superselectively and the dosage of lipiodol and doxorubicin was determined by tumor size, vascularity, presence of, APS and underlying liver function. The embolization endpoint was defined as stasis of blood flow in the tumor-feeding artery, and repeated hepatic arteriography was performed to assess the devascularization after DEM-TACE. If the embolization endpoint was not reached, gelatin sponge particles (Cook, Bloomington, USA), which were mixed with contrast material, were administered into the feeder vessels until stasis in both the DEM-TACE and C-TACE groups.

### Safety assessment of DEM-TACE in PVTT patients

Adverse events (AEs) within 1 months after TACE were performed and reported according to the Society of Interventional Radiology guidelines [16, 17]. Liver function tests after first DEM-TACE were also recorded, such as aspartate aminotransferase, alanine aminotransferase, total bilirubin, serum albumin and prothrombin time, were measured 1 month after the initial TACE procedure to evaluate the safety of DEM-TACE in HCC patients with PVTT.

### Follow-up and re-treatment schedules

All HCC patients in this study were undergone regular follow-up visits after the initial DEM-TACE or C-TACE procedure every 4–8 weeks. Each follow-up visit included a detailed history and physical examination, laboratory tests, and abdominal contrast-enhanced three-phase dynamic spiral CT or MR imaging. Follow-up TACE was repeated when the recurrent or residual tumor was detected by enhanced CT/MR in both groups. And DEM-TACE/C-TACE procedures could be performed in DEM-TACE group and C-TACE group, which depended on tumor burden, prior treatment history and patients’s decision. TKI (sorafenib 400 mg bid) was recommended and administered as per institutional protocol if it was agreed by the patient during the follow-up period, especially in patients diagnosed with PD after the initial TACE procedure. Patients who refused TKI underwent TACE or conservative treatment. Patients were followed up every 2–3 months thereafter. Time to tumor progression (TTP) was defined as the time from the first TACE treatment to progressive disease (PD) according to the modified Response Evaluation Criteria in Solid Tumors (mRECIST) criteria [18]. Overall survival (OS) was defined as the time from the first TACE treatment to death, and patients alive at the end of follow-up were recorded as censored.

### Radiologic response evaluations

Tumor radiologic response was evaluated separately by two radiologist ( both with 10 years of experience in liver imaging) within 4–8 weeks after the initial TACE procedure with contrast-enhanced CT or MR imaging according to the mRECIST criteria: complete response (CR) was defined as the absence of enhanced tumor reflecting complete tissue necrosis in all target lesions; partial response (PR) was defined as at least a 30% decrease in the sum of the diameters of the viable target tumor, reflecting partial tissue necrosis; PD was defined as ≥ 20% increase in the sum of the diameters of viable target tumor or the appearance of any new malignant lesions; and stable disease (SD) was defined as a tumor response between PR and PD [18]. It was also suggested in the mRECIST criteria that the presence of PVTT should be considered as a non-target lesion, and criteria of PVTT radiologic responses include: CR: disappearance of all nontarget lesions; non-CR-non-PD: the persistence of one or more nontarget lesions; and PD is the appearance of one or more new lesions and/or unequivocal progression of existing nontarget lesions [18]. The PVTT radiologic response was thus defined as the percentage of patients who had the tumor response rating of CR, and non-CR-non-PD. Overall response was also evaluated according to the mRECIST criteria [18]. And radiologic response rate was defined as the percentage of patients who showed tumor response level of CR, PR, or SD.

### Statistical analysis

SPSS Statistics®, version 19 (IBM, Armonk, United States), for statistical analysis was used for all analyses. Quantitative data are reported as the mean ± SD and were compared between these two groups using Student’s t-test. Categorical data were compared using the χ2 test. TTP and OS were analyzed using a Kaplan–Meier curve and Breslow test. The Cox proportional hazard model was used for univariate and multivariate analyses to determine prognostic factors. Differences were deemed significant when p < 0.05.

## Results

### Study population

**Fig. 1:**
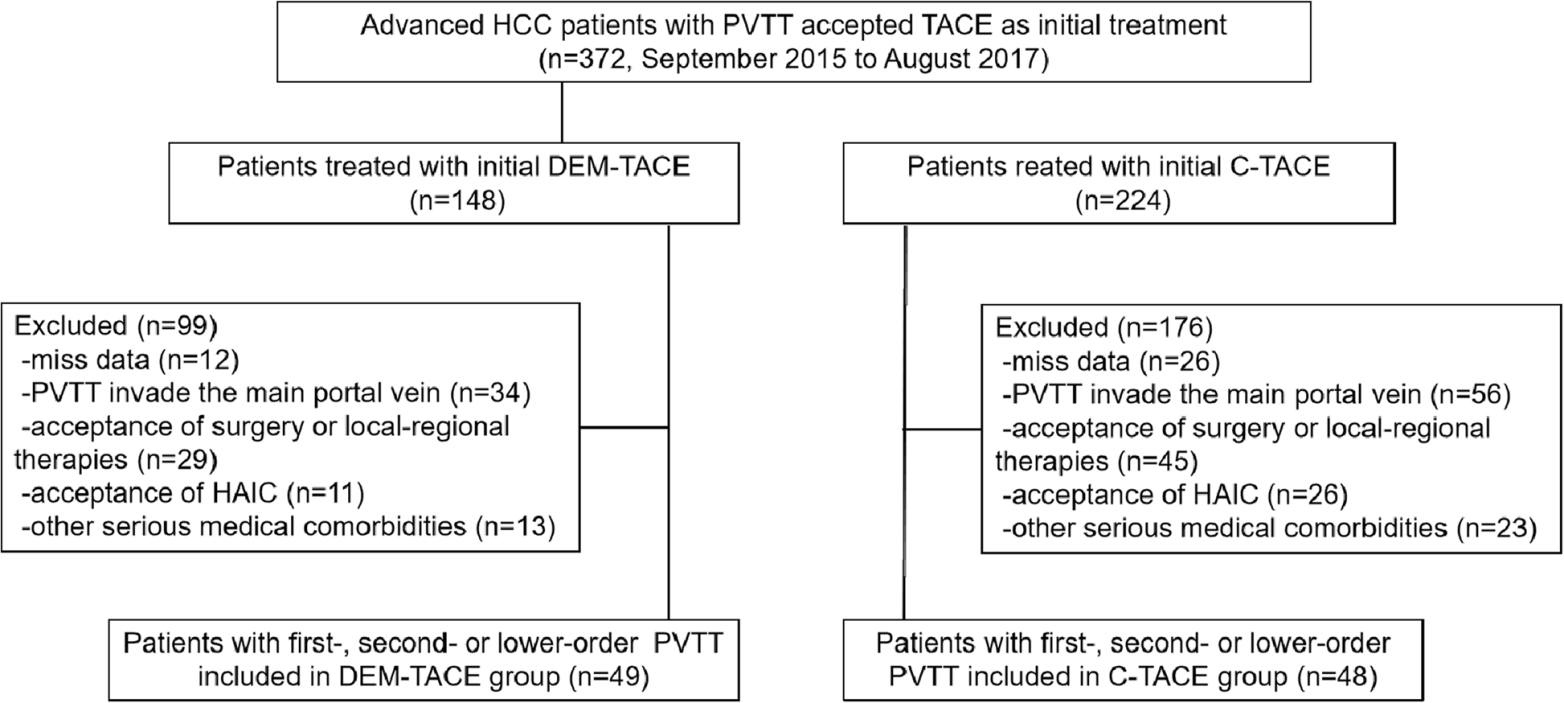
Flow diagram shows exclusion criteria. HCC: hepatocellular carcinoma; PVTT: portal vein tumor thrombus; DEM-TACE: Drug Eluting Microsphere-TACE; C-TACE: Conventional-TACE; HAIC: hepatic arterial infusion chemotherapy

From September 2015 to August 2017, 372 consecutive patients with HCC fulfilling the eligibility criteria were included in this study, 275 patients were excluded: 49 patients were in the DEM-TACE group, and 48 were in the C-TACE group (shown in Fig. 1). The mean follow-up time was 14.9 ± 1.2 (95% CI 12.6–17.2) months ending in May 2019. Table 1 summarizes the baseline characteristics of the study subjects, and there were no significant differences between these two groups for any of the variables. All patients enrolled had BCLC-C with PVTT and large tumors, and 13.4% (14/97) of them had extrahepatic spread at baseline. All TACE procedures were technically successful, with a mean of 3.2 ± 2.1 TACE cycles (DEM-TACE cycles: 1.4 + 0.5; C-TACE cycles: 1.8 + 1.9) in DEM-TACE group and a mean of 3.5 ± 1.7 cycles in C-TACE group (*P* = 0.368). 36 patients in DEM-TACE group had a subsequent C-TACE.

**Table 1:** Baseline characteristics of HCC patients in two groups.^†^ Data are mean ± standard deviation. ^#^ Pearson Chi-Square was used. ^^^ Continuity Correction was used. ^&^ Independent-samples t test was used

	**DEM-TACE (*N* = 49)**	**C-TACE (*N* = 48)**	***P*-Value**
**Mean Age**†	53.67 ± 13.71	51.35 ± 11.34	0.366
**Male: Female**	41 (83.7%): 8 (16.3%)	43 (89.6%): 5 (10.4%)	0.393^#^
**Cause of liver disease**			0.234^^^
**Hepatitis B**	49	45	
**Hepatitis C**	0	0	
**Others**	0	3	
**Child–Pugh score**			0.776^#^
**A**	43 (87.8%)	43 (89.6%)	
**B**	6 (12.2%)	5 (10.4%)	
**ECOG score**			0.247^#^
**0**	36 (73.4%)	30 (62.5%)	
**1–2**	13 (26.5%)	18 (37.5%)	
**a-Fetoprotein level (AFP) (ng/mL)**			0.473^#^
** < 400**	25 (51.0%)	21 (43.8%)	
** ≥ 400**	24 (49.0%)	27 (56.3%)	
**PVTT Stage (1**^**st**^**-order/2**^**nd**^**- or lower order)**			0.118^#^
**1**^**st**^**-order portal vein (group A)**	25 (51.0)	32 (66.7)	
**2**^**nd**^**- or lower order portal vein (group B)**	24 (49.0)	16 (33.3)	
**APS (present/absent)**	17/32	16/32	0.888^#^
**Degrees of APS**[19]			0.231^^^
**0**	6	2	
**1**	4	6	
**2**	5	3	
**3**	2	5	
**Extrahepatic spread**	6 (12.2%)	8 (16.7%)	0.536^#^
**Lymph nodes**	2 (4.1%)	4 (8.4%)	
**Lung**	2 (4.1%)	2 (4.2%)	
**Bones**	1 (2.0%)	1 (2.1%)	
**Suprarenal gland**	1 (2.0%)	1 (2.1%)	
**Tumor size (mm)**†	72.6 ± 20.4	75.1 ± 18.6	0.525^&^
**Tumor Number**			0.356^#^
**Single lesion**	25 (51.0%)	20 (41.7%)	
**Multiple Lesions**	24 (49.0%)	28 (58.3%)	
**TACE Times**	3.2 ± 2.1	3.5 ± 1.7	0.368^&^
**Sorafenib (yes/no)**	5/44	8/40	0.350^#^

### Safety of DEM-TACE vs C-TACE

There was a total of 68 AEs in DEM-TACE group and 86 in C-TACE group without any 30-days mortality nor treatment-related mortality. AEs after the first TACE procedure in both groups are shown in Table 2. DEM-TACE showed a significantly lower incidence of abdominal pain than C-TACE (*P* = 0.032), all other AEs were comparable between the 2 groups (*P* > 0.05). Liver function changes within 1 month after initial DEM-TACE procedures are described in Table 3 and no significant deterioration in liver function in DEM-TACE group.

**Table 2:** Adverse events occurred after initial TACE procedure in both groups. ^#^ Pearson Chi-Square was used. ^^^ Continuity Correction was used

	**DEM-TACE**	**C-TACE**	***P*-Value**
Abdominal plain	21 (42.9%)	31 (64.6%)	0.032^#^
Fever	28 (57.1%)	35 (72.9%)	0.104^#^
Ascites	4 (8.2%)	5 (10.4%)	0.974^^^
Biloma requiring percutaneous drainage	1 (2.0%)	0 (0%)	1.000^^^
Liver abscess	3 (6.1%)	5 (10.4%)	0.689^^^
Spontaneous bacterial peritonitis	4 (8.2%)	4 (8.3%)	1.000^^^
Gastrointestinal hemorrhage/ulceration	2 (4.1%)	1 (2.1%)	1.000^^^
Pulmonary arterial oil embolus	0 (0%)	0 (0%)	-
Iatrogenic artery dissection	1 (2.0%)	2 (4.2%)	0.986^^^
Inguinal hematoma	4 (8.2%)	3 (6.3%)	1.000^^^

**Table 3:** Laboratory test results. There were no significant changes within 1 month before and after DEM-TACE procedure. ALT: alanine aminotransferase, AST: aspartate aminotransferase

	**Baseline**	**1 month after 1**^**st**^** DEM-TACE procedure**	***P*-Value**
ALT	66.6 ± 129.3	58.5 ± 105.3	0.736
AST	66.9 ± 50.9	58.9 ± 69.0	0.515
Total bilirubin level (mmol/L)	17.5 ± 9.7	16.6 ± 9.9	0.657
Serum albumin level (g/L)	39.2 ± 4.2	37.4 ± 4.7	0.055
Prothrombin time (sec)	13.5 ± 1.3	13.8 ± 1.3	0.239

### Assessment of radiologic response

**Fig. 2:**
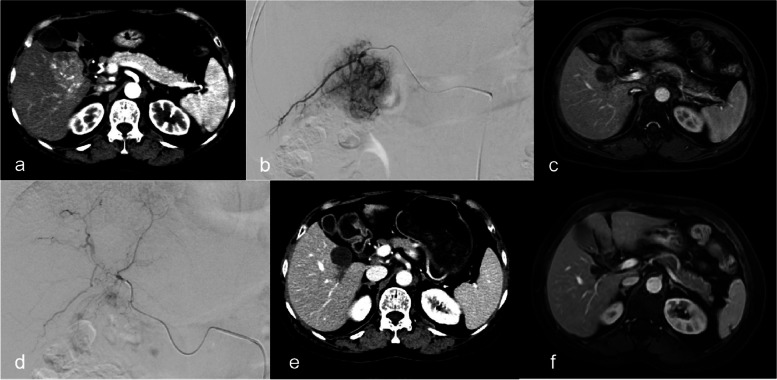
**a**. 66 years old, female patients, contrast enhanced CT cross sectional image demonstrating a HCC lesion in segment 6 and PVTT in the second order portal vein. **b.** the initial DEM-TACE angiogram demonstrate the feeding artery of tumor and PVTT. **c.** Contrast enhanced MRI 1 months after the initial DEM-TACE demonstrate the residual tumor. **d.** second DEM-TACE angiogram demonstrating the residual tumor. **e.** CT images acquired 1 months after the second DEM-TACE demonstrate no viable tumour and PVTT (radiologic response: CR). **f.** MRI images acquired more than 26 months after the second DEM-TACE demonstrate that the disappearance of PVTT and the patent portal vein

The tumor and PVTT radiologic response within 4–8 weeks after inital TACE procedure was recorded (Table 4). In brief, the tumor radiologic response was noted in 44 patients (CR:8, PR:23 and SD:13, 89.8%) in the DEM-TACE group (1 case radiologic response in DEM-TACE group was shown in Fig. 2 a-e) and 36 patients (CR:2, PR:16 and SD:18, 75.0%) in C-TACE group. Meanwhile, the PVTT radiologic response was noted in 42 patients (CR:11, non-CR-non-PD: 31, 85.7%) in the DEM-TACE group and 34 patients (CR:2, non-CR-non-PD: 32, 70.8%) in C-TACE group. DEM-TACE group had better overall response compared to C-TACE group (79.6% vs. 58.3%, *P* = 0.024).

**Table 4:** Tumor and PVTT Radiologic Responses in advanced HCC Patients with PVTT for these Two Groups according to mRECIST. CR: Complete Response; PR: Partial Response; SD: Stable Disease; PD: Progressive Disease

Variable	Tumor Response		Variable	PVTT Response		Overall Response	
	DEM-TACE (*n* = 49)	C-TACE (*n*=48)		DEM-TACE (*n* = 49)	C-TACE (*n* = 48)	DEM-TACE (*n* = 49)	C-TACE (*n* = 48)
**CR (n)**	8	2	**CR (n)**	11	2	7	0
**PR (n)**	23	16	**Non-CR-non-PD, (n)**	31	32	21	16
**SD (n)**	13	18				11	12
**PD (n)**	5	12	**PD (n)**	7	14	10	20
Radiologic Response rate (%)	89.8%	75.0%		85.7%	70.8%	79.6%	58.3%

### TTP and OS and analysis of factors affecting OS

**Fig. 3:**
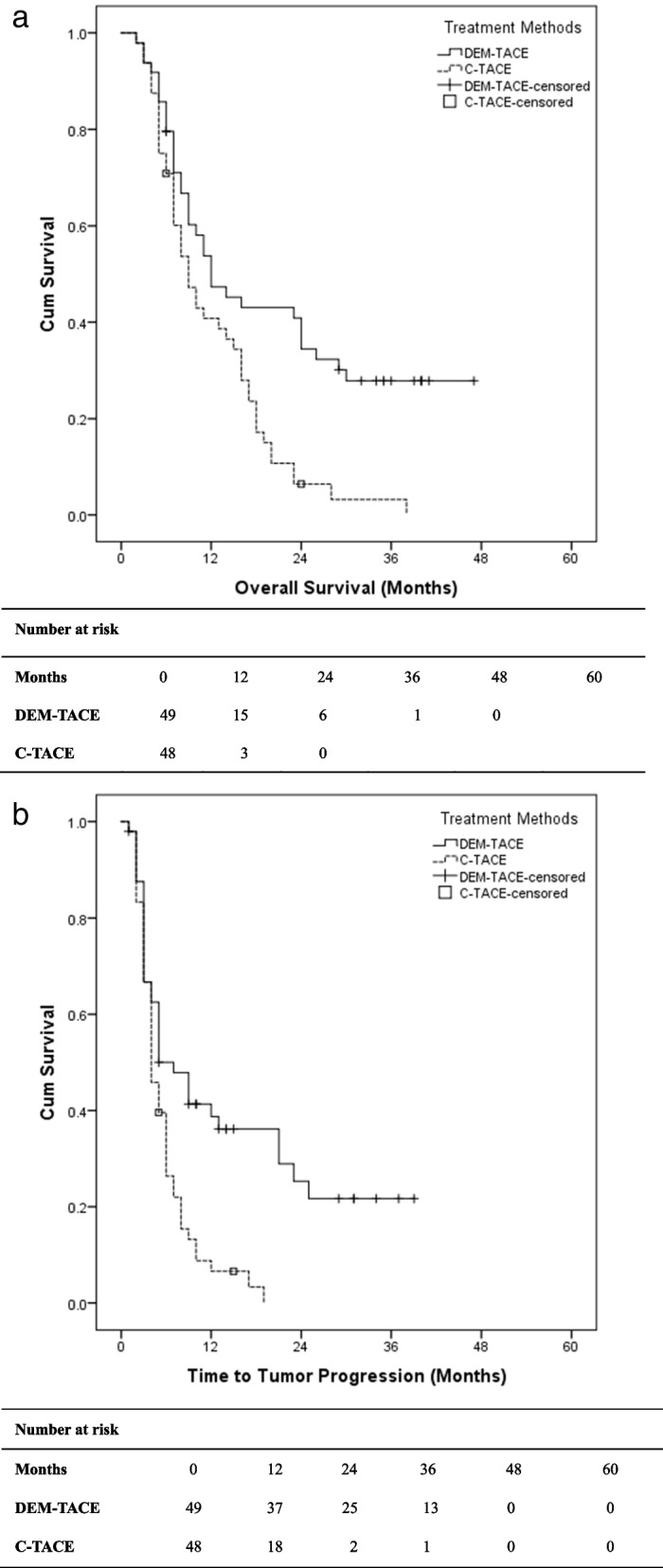
**a**. OS Comparison for DEM-TACE and C-TACE. **b.** TTP Comparison for DEM-TACE and C-TACE. OS: Overall Survival; TTP: Time to Tumor Progression

Both median TTP and OS were found to be superior in the DEM-TACE group compared to the C-TACE group (shown in Fig. 3). The median TTP was calculated to be 7.0 months (95% CI 3.66–10.34) in the DEM-TACE group and 4.0 months (95% CI 2.96–5.04) in the C-TACE group (*P* = 0.040). The median OS was 12.0 months (95% CI 6.32–17.69) in the DEM-TACE group and 9.0 months (95% CI 6.51–11.49) in the C-TACE group (*P* = 0.027). Fifteen (30.6%) patients in the DEM-TACE group and 2 (4.2%) in the C-TACE group were still alive when the analysis was performed (case shown in Fig. 2 f).

The univariate Cox proportional hazards regression model was used to analyze the factors that affect OS. In the univariate analysis, treatment using DEM-TACE, PVTT invading second- or lower-order portal vein branches, absence of APS, encapsulated HCC, smaller tumor diameter, fewer tumor number, AFP value < 400 ng/ml, better tumor radiologic response and PVTT radiologic response were identified as significant factors. All factors with *P*-values less than 0.1 were further included in the multivariate Cox proportional hazards regression analysis, and the results showed that DEM-TACE (*P* = 0.034), absence of APS (*P* = 0.005), AFP value < 400 ng/ml (*P* = 0.019) and better PVTT radiologic response (*P* < 0.001) were independent predictive factors for longer OS. Details are described in Table 5.

**Table 5:** Univariate and multivariate analysis of prognostic factors for OS. OS = Overall Survival; DEM-TACE: drug-eluting microsphere-transarterial chemoembolization; C-TACE: conventional-transarterial chemoembolization; APS: arterioportal shunt; AFP: alpha-fetoprotein; PVTT: portal vein tumor thrombus; CR: Complete Response; PR: Partial Response; SD: Stable Disease; PD: Progressive Disease

		**Univariate Analysis**		**Multivariate Analysis**	
Factors	**No**	**Median OS**	***P*-Value**	**Hazard Ratio**	***P*-Value**
Treatment Method			0.027		0.034
DEM-TACE	49	12.0 (6.3-17.7)		1	
C-TACE	48	9.0 (6.5-11.5)		1.74 (1.04-2.89)	
PVTT Grading			< 0.001		0.385
group A	57	8.0 (6.4-9.6)			
group B	40	23.0 (17.1-28.9)			
APS			< 0.001		0.005
Presence	33	7.0 (5.4-8.6)		1	
Absence	64	16.0 (11.7-20.3)		0.46 (0.27-0.79)	
Encapsulated HCC			0.038		0.074
yes	68	13.0 (8.1-17.9)			
no	29	8.0 (5.5-10.5)			
Maximum tumor diameter (mm)			0.002		0.255
< 5	19	23.0 (14.7-31.3)			
≥ 5	78	9.0 (6.9-11.1)			
Tumor number			0.055		0.403
1-3	45	12.0 (6.5-17.5)			
> 3	52	9.0 (6.8-11.2)			
AFP Value (ng/ml)			0.004		0.019
< 400	46	17.0 (10.5-23.5)		1	
≥ 400	51	8.0 (6.1-9.9)		1.79 (1.10-2.92)	
Tumor radiologic response			< 0.001		0.362
Responders (CR + PR + SD)	80	13.0 (9.1-16.9)			
Non-Responders (PD)	17	7.0 (5.5-8.5)			
PVTT radiologic response			< 0.001		< 0.001
Responders (CR + non-CR-non-PD)	76	16.0 (12.3-19.7)		1	
Non-Responders (PD)	21	5.0 (4.4-5.6)		5.1 (2.58-10.16)	

### Subgroup analysis

**Fig. 4:**
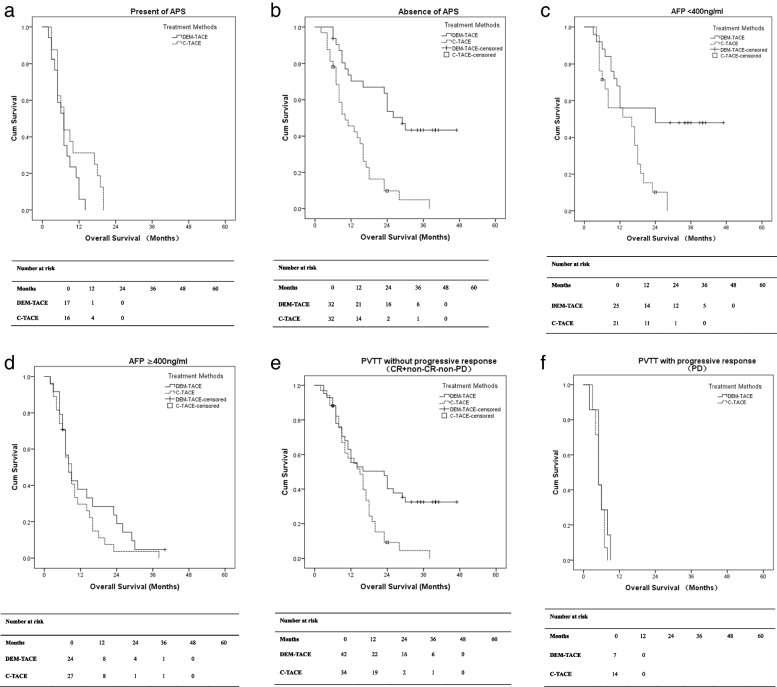
Subgroup analysis of OS: **a,b**: presence/absence of APS; **c,d**: AFP < 400 ng/ml and AFP ≥ 400 ng/ml; **e,f**: PVTT without progressive response/with progressive response. APS: arterioportal shunt; AFP: alpha-fetoprotein; DEM-TACE: drug-eluting microsphere-transarterial chemoembolization; C-TACE: conventional-transarterial chemoembolization; PVTT: portal vein tumor thrombus; CR: Complete Response; PD: Progressive Disease

Subgroup analyses on patients who demonstrated APS during angiography study showed that the median OS of patients was 7.0 months (95% CI 5.07–8.93) in DEM-TACE group and 7.0 months (95% CI 5.06–8.95) in C-TACE group (*P* = 0.095). In patients with absence of APS, the median OS were 29.0 months (95% CI 22.86–35.15) in DEM-TACE group and 10.0 months (95% CI 4.60–15.40) in C-TACE group (*P* < 0.001) (shown in Fig. 4a, 4b).

The median OS of patients with AFP < 400 ng/ml was 24.0 months in DEM-TACE group and 16.0 months (95% CI 6.38–25.62) in C-TACE group (*P* = 0.004). In patients with AFP ≥ 400 ng/ml, the median OS was 8.0 months (95% CI 5.06–10.94) in DEM-TACE group and 9.0 months (95% CI 6.50–11.50) in C-TACE group (*P* = 0.255) (shown in Fig. 4c, 4d).

In patents with PVTT without progressive response (CR + non-CR-non-PD), the median OS was 23.0 months (95% CI 10.70–35.30) in DEM-TACE group and 15.0 months (95% CI 10.33–19.67) in C-TACE group (*P* = 0.004). In patients with PVTT with progressive response (PD), the median OS was 5.0 months (95% CI 2.43–7.57) in DEM-TACE group and 5.0 months (95% CI 4.09–5.91) in C-TACE group (*P* = 0.380) (shown in Fig. 4e, 4f).

## Discussion

Advanced HCC patients with PVTT are not an absolute contraindication to C-TACE according to the consensus-based clinical practice guidelines [20, 21], and several studies suggested that C-TACE could prolong OS in HCC patients with PVTT [22, 23]. However, the tumor response of C-TACE might be unsatisfactory, which might be due to (1) tumor burden and lipiodol retention in large tumor(s) with PVTT and (2) the presence of APS (28.8% to 63.2% in HCC cases) in PVTT patients, which might cause the oil emulsion used in C-TACE to enter the portal vein through the shunt, resulting in hepatic infarction [24, 25]. DEM-TACE is a relatively new technology commonly used in BCLC-B HCC treatment, but its significance in advanced stage HCC with PVTT has not yet been reported.

Several reports suggested that DEM-TACE was well tolerated in HCC treatment, and it was reported that it could be safely performed in advanced HCC patients [26]. Our study reported a comparable complication rate in both C-TACE and DEM-TACE, except for abdominal pain incidence (DEM-TACE: 21 vs. C-TACE: 31, *P* = 0.032). Both treatments could be safely performed in the treatment of advanced HCC without 30-day mortality.

It has been previously suggested that DEM-TACE has better tumor response than C-TACE in several studies [27, 28]. Seki et al. used HepaSphere microspheres and achieved a CR of 12.6% and a PR of 43.7%, which are similar to our results (CR: 16.3% and PR 46.9%) [29]. Our data were consistent with previous studies showing that the overall radiologic response in the DEM-TACE group was better than that in the C-TACE group, which was indicated to be an independent predictive factor for longer OS in other study [30]. Meanwhile, PVTT radiologic response after DEM-TACE have been first reported in this study, which was also better than that in C-TACE group (85.7% vs. 70.8%, *P* = 0.022), which was indicated to be an independent predictive factor for longer OS in our multivariate analysis (*P* < 0.001). Furthermore, in the current study, DEM-TACE was proven to prolong the TTP (7.0 months (95% CI 3.66–10.34) vs 4.0 months (95% CI 2.96–5.04), *P* = 0.040). Our study group inferred the following from the current findings contributing to the superior efficacy of DEM-TACE: first, the feature of DEM-TACE in terms of its conformity allows deeper penetration into the feeding artery of the tumor and PVTT, which might lead to occludes vessels effectively [31]. Previous histologic examination also shown that HepaSphere microspheres could be found in the PVTT without recanalization [32]. Second, the slow release of anticancer drugs from DEM-TACE in HCC enables a sustained anti-tumor effect [33]. These data suggested that initial DEM-TACE might induce extensive intrahepatic tumor/PVTT necrosis compared with C-TACE, which may improve local tumor control and prolong the TTP.

In the present study, DEM-TACE was shown to prolong the OS compared to C-TACE in advanced HCC patients with PVTT (12.0 months (95% CI 6.32–17.69) vs 9.0 months (95% CI 6.51–11.49), *P* = 0.027), which has been rarely reported in previous study. Additionally, our comparison data along with our multivariate analysis suggested that treatment with initial DEM-TACE was an independent predictive factor for longer OS, which has seldom been reported. This might be due to patient selection, as most of the previous reports were mainly in treating intermediate stage HCC. Patient selection is key to optimal cancer treatment, and in our study, we demonstrated the significance of DEM-TACE for BCLC-C patients with PVTT. Fifteen (30.6%) patients treated with DEM-TACE were still alive at the end of the study, meaning that the OS of some patients was over 2 years. These data also echoed a recent retrospective subgroup analysis [28]. Further study should explore prospectively in a multi-center setting to confirm the importance of this advanced disease and compare it with TKI therapy alone, which is widely recommended for BCLC-C patients with PVTT.

Other independent predictive factors for better OS shown in our multivariate analysis are the absence of APS and lower AFP value. APS had previously been reported to be correlated with the poor response of TACE in advanced HCC patients because embolic lipiodol of C-TACE may be diverted into the portal vein branches and delivered to nontumor hepatic tissue instead of being deposited intratumorally as reported in several studies [22, 34]. However, subgroup analysis showed that there was no significant difference in OS between DEM-TACE and C-TACE patients with APS in this study (7.0 months vs 7.0 months, *P* = 0.095). We assumed that there might be two reasons: first,patients with severe APS (classification 1–3: 11 patients in DEM-TACE group and 14 patients in C-TACE group) might be not suitable for TACE procedure, even when using the HepaSphere microspheres, which might pass through the shunt during TACE[35]. Meanwhile the number of patients with APS (17/49 in DEM-TACE group and 16/48 in C-TACE group) was limited which could also have led to this result in subgroup analysis. Meanwhile, DEM-TACE was proven to gain longer OS compared to C-TACE with better PVTT response in (CR + non-CR-non-PD) subgroup analysis. These data indicated that despite not hereby directly supported we cannot rule out the possibility that APS management with DEM-TACE could potentially achieve superior radiologic response, which is essential to achieve better outcomes [36]. AFP value is thought to be associated with tumor activity and to play an important role in the degree of HCC malignancy in cytologic studies [37]. This study showed that higher AFP level was a poor prognostic factor for OS, which was also proven in our previous study [38]. Our data also align with previous findings and provide a guide for better patient selection for DEM-TACE treatment in advanced HCC patients.

Limitations of this study include the following: first, retrospective, and therapeutic options (DEM-TACE vs. C-TACE) in advanced HCC patients with PVTT were individually determined by the patients’ preference, which likely led to selection bias in our population. However, the bias was limited by similar baseline characteristics between these two groups. Second, the number of patients was relatively small (49 patients in DEM-TACE and 48 patients in C-TACE), but it was sufficient to demonstrate the significance in terms of radiologic response, TTP and OS, as shown in our statistical analysis and sample size estimation. Third, most HCC tumors were not histopathologically confirmed but were diagnosed based on imaging and AFP level as per the EASL guidelines. Fourth, TKIs are recommended as the standard of care for advanced HCC patients [19], and it was proven that the combination with TACE could improve OS in advanced HCC patients with PVTT in our previous study [39]. However, only a small number of patients (5 in the DEM-TACE group and 8 in the C-TACE group, *P* = 0.350) had TKIs in this study. TKI combined with DEM-TACE is worth further exploration. Fifth, in the patients’ baseline characteristics, higher percentage of 1st order PVTT (66.7%) and multiple tumors (58.3%) reported in C-TACE group as compared to 51.0% and 49.0% in DEM-TACE group, respectively. Although there was no statistical significance, it might affect clinical outcomes. Lastly, this result was only reported from a single center, and a multicenter clinical trial is suggested for further verification.

## Conclusion

DEM-TACE yielded promising efficacy outcomes in HCC patients with PVTT and is a potential option for this advanced disease. Patients treated with initial DEM-TACE showed better response and longer TTP/OS than patients treated with initial C-TACE. A multicenter prospective randomized controlled trial is suggested to show differences in local tumor control and survival of advanced HCC patients.

## Data Availability

The datasets used and/or analysed during the current study are not publicly available due to data privacy ordinance but are available from the corresponding author on reasonable request.

## Abbreviations

HCC: Hepatocellular carcinoma
PVTT: Portal vein tumor thrombus
BCLC: Barcelona Clinic Liver Cancer
TKI: Tyrosine kinase inhibitor
ICIs: Immune checkpoint inhibitors
C-TACE: Conventional transarterial chemoembolization
DEM-TACE: Drug-eluting microsphere-TACE
EASL: European Association for the Study of the Liver
ECOG: Eastern Cooperative Oncology Group
APS: Arterioportal shunt
AEs: Adverse events
TTP: Time to tumor progression
OS: Overall survival
mRECIST: Modified Response Evaluation Criteria in Solid Tumors
PD: Progressive disease
CR: Complete response
PR: Partial response
SD: Stable disease
